# HISLIS: Histology, Sarcopenia, and Lung Inflammation Score—A New Perspective for Lung Cancer Patients?

**DOI:** 10.3390/life15060963

**Published:** 2025-06-16

**Authors:** Claudia Raluca Mariean, Oana Mirela Tiucă, Cristina Flavia Al-Akel, Ovidiu Simion Cotoi

**Affiliations:** 1 Doctoral School of Medicine and Pharmacy, George Emil Palade University of Medicine, Pharmacy, Science, and Technology of Targu Mures, 540142 Targu Mures, Romania; 2 Pathophysiology Department, George Emil Palade University of Medicine, Pharmacy, Science, and Technology of Targu Mures, 540142 Targu Mures, Romania; 3 Department of Radiology, Targu Mureș County Emergency Hospital, 540142 Targu Mures, Romania; 4 Dermatology Department, George Emil Palade University of Medicine, Pharmacy, Science, and Technology of Targu Mures, 540142 Targu Mures, Romania; 5 Dermatology Clinic, Mures Clinical County Hospital, 540342 Targu Mures, Romania; 6 Department of Pediatric Cardiology, Emergency Institute for Cardiovascular Diseases and Transplantation of Târgu Mureș, 540136 Targu Mures, Romania; 7 Pathology Department, Mures Clinical County Hospital, 540011 Targu Mures, Romania

**Keywords:** lung cancer, histologic subtype, sarcopenia, CBC-derived systemic inflammation, computed tomography, HISLIS, severity grade

## Abstract

Background: Since lung cancer remains a health problem worldwide and is the leading cause of cancer death, finding new tools that can help in the early identification of high-risk patients remains a key target. Methods: A retrospective descriptive study of 70 patients diagnosed with lung cancer at the Clinical County Hospital Mureș was conducted. Information regarding the histopathological type of the tumor, the TNM stage at diagnosis, and the CBC-derived inflammatory status was obtained for all the included patients. Skeletal muscle area was measured at the level of the third lumbar vertebra (L3SMA), based on the patients’ native CT scans, to identify sarcopenia. These four primary characteristics (the histopathological type of the tumor, the TNM stage, the systemic inflammatory status, and the sarcopenic changes) were integrated into a new severity score: the histology, sarcopenia, and lung inflammation score (HISLIS). Subsequently, based on the HISLIS score, the patients were divided into three severity grades (high, medium, and low). Results: Our results showed that patients diagnosed in late advanced TNM stages (III or IV) had the highest severity grade. The severity grade strongly correlated with the systemic inflammatory biomarkers, with the highest severity grades being associated with an increased inflammatory status. In addition, sarcopenic patients were diagnosed in more advanced TNM stages, exhibited higher HISLIS levels, and had a higher degree of systemic inflammation than non-sarcopenic patients. Sarcopenic patients also showed an impaired hematological profile, with hemoglobin (Hb) and hematocrit (Ht) levels being significantly decreased in sarcopenic patients. Conclusions: Future prospective studies are needed to validate the HISLIS and integrate it into the routine clinical and paraclinical assessment of lung cancer patients, as it could represent a triage tool for the early identification of patients at higher risk of unfavorable outcomes. Combining critical information regarding the tumors’ characteristics, such as TNM stage and histological characteristics, together with biological and imaging-derived information like the CBC-derived inflammatory status and the associated degree of sarcopenia, could lead to a complex approach and a personalized therapeutic regimen for this highly deadly condition.

## 1. Introduction

Lung cancer is the second most frequently diagnosed malignancy in both men and women and is the leading cause of cancer death worldwide. Lung cancer patients are frequently diagnosed in the late, advanced stages of the disease, when treatment regimens are barely curative [1]. Men are affected more frequently than women and have a lower 5-year survival rate (19% versus 27%), partly because women are diagnosed more frequently at a localized stage than men are (28% versus 23%). Women are more likely to have associated genetic mutations in their tumors; therefore, they are more sensitive to target therapies [2,3].

Although up to 80% of lung cancer patients are smokers, there are other associated risk factors related to lung carcinogenesis, such as genetic factors, ambient and indoor air pollution, and exposure to toxic substances like radon gas, arsenic, and benzene [4,5]. In addition, previous studies have shown that a history of lung disorders such as pneumonia, asthma, chronic obstructive pulmonary disease (COPD), tuberculosis, chronic bronchitis, and pulmonary fibrosis is associated with an increased risk of lung cancer [6,7,8].

Lung cancer can be divided into two main histopathological types: small cell lung carcinoma (SCLC) and non-small cell carcinoma (NSCLC), which accounts for up to 85% of cases. NSCLC can be divided into adenocarcinoma, which is the most common pathological subtype, squamous cell carcinoma, and large cell carcinoma [9]. Different histological subtypes of lung carcinoma have different characteristics and prognoses. Squamous cell carcinoma only responds to surgical interventions and is insensitive to radiotherapy and chemotherapy. In contrast, due to frequently associated genetic mutations like epidermal growth factor receptor (EGFR) mutation, adenocarcinoma patients have a better treatment response and improved progression-free survival rates [10,11,12]. SCLC has the worst prognosis and the highest degree of malignancy [5].

Sarcopenia, a skeletal muscle disease, involves a progressive loss of muscle mass and function [13,14]. Sarcopenic changes can be either due to aging or can be associated with various pathologic conditions, such as respiratory, cardiac, and cognitive disorders, as well as different malignant conditions. Sarcopenia increases the risk of functional impairment, length of hospitalization, and mortality rates [15,16]. Recent studies published by Collins et al. [17] and Nishimura et al. [18] observed a loss of muscle mass and body weight in lung cancer patients. Sarcopenia involves a poor prognosis for lung cancer patients, partially due to the presence of tumor-secreted myokines [19,20]. As sarcopenic changes impact clinical evolution, an early diagnosis of sarcopenia could improve the clinical outcome and the overall survival of these patients.

Nowadays, sarcopenia can be diagnosed based on bioelectrical impedance analysis (BIA), dual-energy X-ray absorptiometry (DXA), and computed tomography scans (CT scans). In recent years, the use of CT scans as diagnostic tools for sarcopenia has increased; this imaging method represents a rapid and inexpensive technique that can offer crucial clinical information by measuring the total skeletal muscle area (SMA). An international consensus chose SMA measurements at the L3 level (L3SMA) as reference values, as they reflect the overall muscle mass of the patients [21]. L3SMA is obtained by summing the skeletal muscle areas of the psoas, paraspinal, and abdominal wall muscles, with both transverse processes visible [22].

Besides the sarcopenic changes, cancer patients have an associated degree of inflammation, which promotes, up to a certain point, the malignant cells’ proliferation, angiogenesis, and metastases by influencing the tumor microenvironment and the overall immune response. Although studies have discussed the importance of various cytokines, interleukins, and specific immune molecules in cancer-associated processes, such specific and expensive laboratory tests are not performed in a patient’s routine clinical and paraclinical assessment at admission. Therefore, as the analysis of a routinely performed examination like the complete blood count (CBC) can offer important information about the patient’s systemic inflammatory status and is easily achievable for all cancer patients, analyzing the CBC has garnered a lot of interest in the scientific world. CBC-derived inflammatory biomarkers like the neutrophil–lymphocyte ratio (NLR), platelet–lymphocyte ratio (PLR), monocyte–lymphocyte ratio (MLR), the systemic inflammation response index (SIRI), the aggregate index of systemic inflammation (AISI), and the systemic immune inflammation index (SII) can be used as prognostic factors in various malignancies, including those in both NSCLC and SCLC patients [23,24,25,26]. Nevertheless, the association between inflammatory changes and the presence of sarcopenic changes can be made in the case of cachectic patients. The definition of cachexia was updated in 2019 by Cederholm et al., who published a revised definition of it based on a consensus report from the Global Clinical Nutrition Community [27]. In this updated definition, cachexia was described as chronic disease-related malnutrition with associated inflammatory changes. One phenotypic criterion among involuntary weight loss, low muscle mass, and low BMI, and one etiologic criterion among reduced food assimilation/intake or associated inflammatory changes are needed to diagnose cachexia [27,28].

In light of all the aspects mentioned above, our study aimed to offer a complex perspective regarding a population of lung cancer patients in which the inflammatory and sarcopenic changes were analyzed and integrated with the histopathological type of the tumor and its TNM stage at the time of initial diagnosis to develop a new prognostic severity score. We named the proposed score HISLIS: the histology, sarcopenia, and lung inflammation score. By incorporating sarcopenia and inflammation as central variables, the model aims to provide additional insights into the complexity of patients’ biological status and may support a personalized therapeutic approach for lung cancer patients. The HISLIS could be used as a triage tool for identifying oncology patients at increased risk of unfavorable outcomes, aiming to serve as a primary step in the complex evaluation of all the key findings that can influence the proper approach, diagnosis, and management of this highly deadly condition.

## 2. Materials and Methods

We present the results of a retrospective descriptive study with a cohort of 70 patients diagnosed with lung carcinoma between 1 January 2019 and 31 December 2023 at the Clinical County Hospital Mures, Târgu Mures, Romania.

The following criteria were used as inclusion criteria: (1) a histopathological diagnosis of lung carcinoma, (2) selected laboratory data that were available for analysis, (3) a CT scan that included the L3 vertebra, (4) patients aged >18 years old, or (5) patients without associated active infections or any other type of malignancies at the time of diagnosis.

The exclusion criteria included the following: (1) patients without a histopathological confirmation of the diagnosis of lung carcinoma, (2) patients without the selected laboratory data needed for analysis, (3) patients without a CT scan that included the L3 vertebra, (4) patients aged <18 years old, or (5) patients with associated active infections or other associated malignancies at the time of diagnosis.

This study was performed according to the Declaration of Helsinki and was approved by the Ethics Committee of the Clinical County Hospital Mures (approval 20419/15 December 2023).

### 2.1. Definition of the Analyzed Parameters

The analyzed parameters included the following:

- The tumor stage at diagnosis: the patients’ tumor stage was established, considering the TNM classification of malignant tumors. In this classification, T describes the primary tumor size and site, N describes the involvement of the regional lymph nodes, and M represents the presence of distant metastasis. In our study group, the 8th edition of the TNM grading system was used to determine the proper tumor staging of the patients [26].
- The histological type of lung carcinoma: NSCLC (adenocarcinoma, squamous cell carcinoma, adenosquamous carcinoma, NSCLC that is not otherwise specified (NOS)), and SCLC.
- Sarcopenia was assessed, using the CT scans that were performed at the time of initial diagnosis. For this purpose, we used the ODIASP software tool (2.2.9), which automatically detected and calculated the skeletal muscle cross-sectional area (SMA) at the third lumbar vertebra level (L3) [29,30,31,32]. Patients were classified as sarcopenic if their SMA values were below the following cut-off values described in the literature: 92.2 cm in females and 144.3 cm in males [33].
- The inflammatory status of the patients at the time of initial diagnosis was assessed based on the analysis of parameters derived from their CBCs. The following cellular lines, similar to CBC-derived inflammatory indexes, were calculated and analyzed for each patient: leucocyte count, neutrophil count, lymphocyte count, monocyte count, eosinophil count, and platelet count, used to calculate the neutrophil-to-lymphocyte ratio (NLR), derived neutrophil-to-lymphocyte ratio (d-NLR), monocyte-to-lymphocyte ratio (MLR), platelet-to-lymphocyte ratio (PLR), eosinophil-to-neutrophil ratio (ENR), eosinophil-to-monocyte ratio (EMR), systemic inflammatory index (SII), systemic inflammatory response index (SIRI), and aggregate index of systemic inflammation (AISI).

The detailed formulas of the included CBC-derived inflammatory indexes are displayed in Table 1.

- A prognostic severity score—the HISLIS: histology, sarcopenia, and lung inflammation score—was calculated for each patient. Sarcopenia and CBC-derived inflammatory biomarkers were assessed as binary variables. Patients were awarded 1 point if sarcopenia was present or if the CBC-derived inflammatory biomarkers were increased above the threshold values. The TNM stage was evaluated as an ordinal variable, with patients being awarded points from 0 to 3, depending on the TNM stage at diagnosis. The histological subtype was also considered and graded based on pre-existing literature data about the associated severity of each histological subtype [5,10,11,12]. The total prognostic score was obtained by summing the points assigned to each clinical and inflammatory predictor according to the predefined scheme.

The detailed methodology for constructing the HISLIS score is presented in Table 2, as seen below.

- Severity grades of the prognostic HISLIS score: the final score was divided into three severity grades, using percentile-based thresholds to enable clinically practical interpretation and risk stratification. Based on the empirical distribution of scores within the analyzed cohort, the patients were stratified into three severity grades (low, intermediate, and high). The classification was performed using the 33rd and 66th percentiles (P33 and P66) to provide an objective stratification of patients without relying on arbitrary thresholds and to reflect the natural distribution of the score in our study population. The score thresholds were defined as follows:
  ✓ Score < P33 (below 8.42): Low severity
  ✓ Score between P33 and P66 (8.42–10.00): Intermediate severity
  ✓ Score > P66 (above 10.00): High severity
- BMI was calculated using the following formula: BMI = kg/m^2^. Based on their BMI, patients were classified as underweight (BMI < 18.5 kg/m^2^), normal weight (BMI between 18.5 and 24.99 kg/m^2^), overweight (BMI between 25 and 29.99 kg/m^2^), grade I obesity (BMI between 30 and 34.99 kg/m^2^), grade II obesity (BMI between 35 and 39.99 kg/m^2^), and grade III obesity (BMI > 40 kg/m^2^).
- Data regarding the exposure to tobacco smoke, the presence of COPD as a comorbidity for the current disease, living environment (urban/rural), the gender, and the age of the patients at diagnosis were also analyzed.

### 2.2. Statistical Analysis

The statistical analysis of our study population was made with the aid of JASP Team (2024) software from JASP (version 0.19.3). Descriptive statistics were used to characterize the study population regarding their sarcopenia status, the histological subtype of the lung carcinoma, the TNM stage, and the CBC-derived inflammatory biomarkers at diagnosis. Spearman’s rank correlation analysis explored the relationship between systemic inflammatory markers, SMA values, the HISLIS severity score, and additional significant clinical parameters. The Mann–Whitney U test was applied to compare continuous variables between patients with and without sarcopenia. The Kruskal–Wallis test was applied to identify significant differences in tumor stage and inflammatory indices across severity grades. Ordinal logistic and multivariate regression analyses were used to identify the independent predictors of increased severity grade.

## 3. Results

### 3.1. Characterization of the Study Population

#### 3.1.1. General Characteristics

The study included 70 patients diagnosed with lung carcinoma, 53 of whom were males and 17 of whom were females. The median age at diagnosis was 67.3 years. Regarding the living environment, most patients lived in rural areas (37 versus 13 patients).

**Figure 1:**
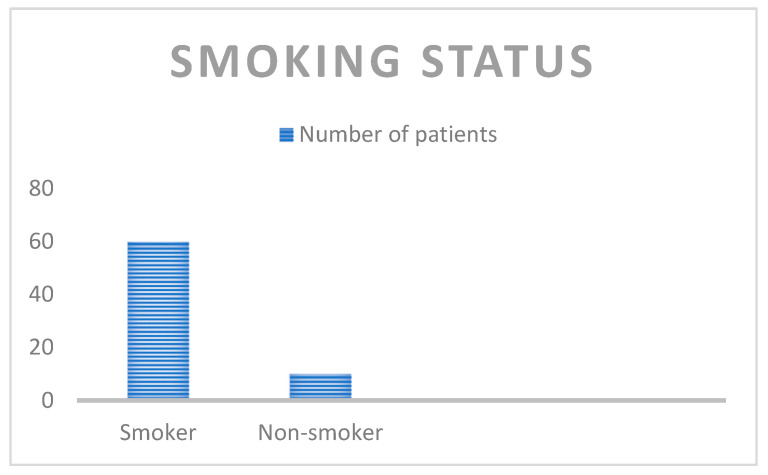
Smoking status of the study population.

Figure 1 depicts the smoking status, as 60 of the included patients were smokers, while only 10 patients declared that they did not smoke.

#### 3.1.2. The Histological Type of the Tumor

Of the 70 patients in the study population, 6 were diagnosed with SCLC, while 64 were diagnosed with NSCLC. Of the 64 patients diagnosed with NSCLC, 33 were diagnosed with adenocarcinoma, 25 were diagnosed with squamous cell carcinoma, and 6 were diagnosed with other types of NSCLC (adenosquamous and NOS carcinoma).

**Figure 2:**
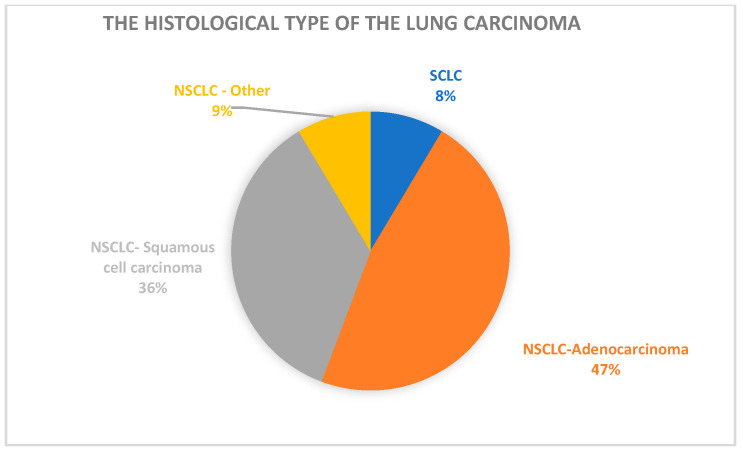
Histopathological classification of lung cancer types in the cohort.

Figure 2 visually represents the histological characteristics of the study population.

#### 3.1.3. The TNM Stage at Diagnosis

Out of the 70 patients included, most were diagnosed in late-advanced stages. Thirty-nine patients were diagnosed at stage IV, twenty-nine at stage III, and only two at stage II. No patient was diagnosed at stage I.

**Figure 3:**
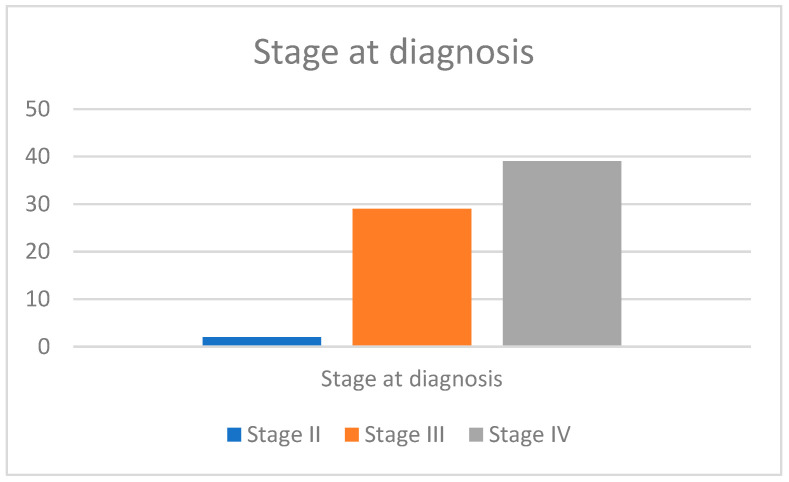
Stage at diagnosis of the study population.

The graphical representation of the presented data is found in Figure 3.

### 3.2. The Sarcopenia Assessment and Its Impact on the Study Population

Sarcopenia was assessed at the time of initial diagnosis, based on the SMA measured at the level of the L3 vertebra, using the ODIASP software. Sarcopenic changes were present in 34 out of 70 patients at the initial diagnosis.

**Figure 4:**
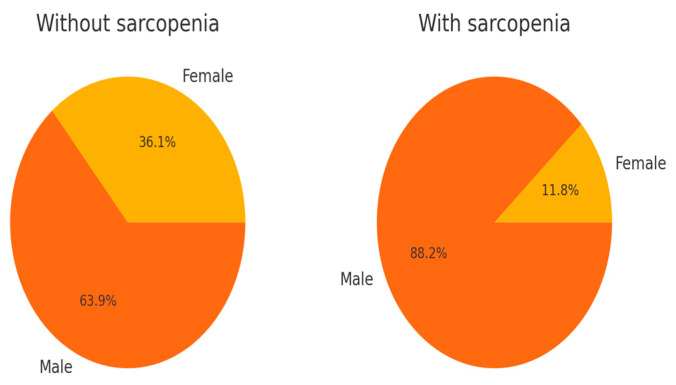
Sarcopenia distribution based on the gender of the study population.

The exact distribution of sarcopenic changes, based also on the gender of the patients, is depicted in Figure 4.

#### 3.2.1. Association Between Sarcopenic Changes and Clinical and Biological Parameters

We explored whether sarcopenia significantly correlates with the included patients’ routine clinical and biochemical parameters. Therefore, Mann–Whitney U tests were applied to all numeric variables of interest. Our results showed that patients with sarcopenia had significantly lower levels of hemoglobin (Hb) (*p* = 0.0162) and hematocrit (Ht) (*p* = 0.0147) than non-sarcopenic patients, suggesting a potential relationship between sarcopenia and anemia. In addition, we observed that sarcopenic patients were significantly older than non-sarcopenic patients (*p* = 0.0082).

#### 3.2.2. Association Between SMA and Clinical and Biological Parameters

Spearman’s rank correlation analysis was performed to evaluate the relationship between the exact value of SMA and relevant clinical and biological parameters. The results showed a significant negative correlation between SMA and tumor stage (ρ = −0.33, *p* = 0.0037), indicating that skeletal muscle mass tends to decrease as the disease advances. Conversely, positive correlations were observed between SMA and both hemoglobin (Hb) (ρ = 0.31, *p* = 0.0052) and hematocrit (Ht) (ρ = 0.29, *p* = 0.0109), reinforcing the idea that a higher muscle mass is associated with improved hematologic profiles in lung cancer patients.

### 3.3. The Inflammatory Profile of the Study Population

#### 3.3.1. General Considerations

The study’s next step was to investigate the inflammatory profile of the study population at the time of initial diagnosis. Therefore, we compared the inflammatory response of the included patients with the reference values cited in the literature. The following reference values were used: 265 Leukocytes ×10^3^/μL: 4.1–12.2; Neutrophils × 10^3^/μL 1.50–7.90; Lymphocytes ×10^3^/μL: 1.10–3.40; Monocytes ×10^3^/μL: 0.30–1.10; Eosinophils ×10^3^/μL: 0.00–0.50; Platelets ×10^3^/μL: 150–400; NLR: 0.78–3.53 (13); d-NLR: 268 <= 2.2; MLR: 0.352–0.369; PLR: <185; EMR: 0.02–0.24; ENR: < 0.05; SII: 660 (NSCLC)/1600 (SCLC); AISI: 351; SIRI: 2;

Table 3 presents the number of patients with elevated inflammatory markers and cellular line count values, based on the disease stage at diagnosis.

The results revealed a progressive increase in the frequency of elevated values with advancing stage for most parameters. For instance, AISI was elevated in nearly all stage IV patients (36/39) and in most stage III cases (23/29), while only two patients in stage II exhibited elevated values. Similar patterns were observed for SIRI (elevated in 33/39 patients at stage IV), SII, and NLR values. These showed an increase in the numerical distribution of patients with increased inflammatory status as the TNM stage advanced, indicating a strong association between tumor progression and systemic inflammation.

Cell line disturbances followed a similar trend: leukocytosis, neutrophilia, and thrombocytosis were predominantly observed in stage IV patients, while very few abnormalities were detected in stage II.

#### 3.3.2. Assessment of the Relationship Between Systemic Inflammatory Markers and Clinical Parameters

Spearman’s rank correlation analysis was conducted to explore the relationship between the systemic inflammatory markers and relevant clinical parameters. The analysis included all the inflammatory parameters cited in Table 1. Our study showed that SII showed a statistically significant negative correlation with age (ρ = −0.26, *p* = 0.0268) and BMI (ρ = −0.30, *p* = 0.0277) and was positively correlated with tumor stage (ρ = 0.24, *p* = 0.043). Additionally, AISI was negatively correlated with BMI (ρ = −0.28, *p* = 0.0445).

### 3.4. The Severity Score and Its Global Impact

Our results showed that the majority of patients who exhibited a high severity grade were patients with a stage IV diagnosis of lung cancer. In contrast, few patients with stage IV cancer experienced a low or a medium grade of severity, as seen in Table 4.

#### 3.4.1. Correlation Analysis Between Severity Grade and Clinico-Biological Variables

Spearman’s rank correlation analysis was performed to identify the strongest associations between severity grade and key clinical and inflammatory parameters.

The results revealed that the strongest correlations were found between severity grade and PLR (ρ = 0.74, *p* < 0.001), NLR (ρ = 0.70, *p* < 0.001), SIRI (ρ = 0.62, *p* < 0.001), and MLR (ρ = 0.61, *p* < 0.001) levels.

#### 3.4.2. Parameters That Influence the Degree of Severity

The Kruskal–Wallis test was applied to assess whether the distribution of selected clinical and inflammatory variables differed significantly across the three predefined severity grades (low, medium, and high).

The results indicated statistically significant differences for several parameters. The tumor stage showed a significant association with severity grade (Chi^2^ = 8.8, *p* = 0.0123), suggesting a progressive increase in clinical stage with rising severity. Additionally, all evaluated inflammatory markers: NLR (Chi^2^ = 37.74, *p* < 0.0001), d-NLR (Chi^2^ = 20.33, *p* < 0.0001), MLR (Chi^2^ = 27.76, *p* < 0.0001), PLR (Chi^2^ = 40.73, *p* < 0.0001), SII (Chi^2^ = 16.12, *p* = 0.0003), AISI (Chi^2^ = 25.45, *p* < 0.0001), and SIRI (Chi^2^ = 32.2, *p* < 0.0001) were more elevated in patients with higher grades of severity than in those with lower grades of severity.

### 3.5. Main Differences Between Sarcopenic and Non-Sarcopenic Patients

The last step of our study was a comparative analysis of the differences encountered in the clinical and biological characteristics of patients with and without sarcopenia.

The results showed that patients with sarcopenia had a more advanced tumor stage (median: 4.0 vs. 3.0; *p* = 0.0104), older age (median: 70.0 vs. 65.0; *p* = 0.0177), and a higher severity score (median: 11.0 vs. 9.0; *p* = 0.0038) compared to those without sarcopenia. The results are given in Table 5.

Although not statistically significant, trends were also observed toward higher values of inflammatory markers such as NLR, d-NLR, PLR, SII, and SIRI in the sarcopenic group. Despite a slightly lower median in the sarcopenic group, no significant differences were found in body mass index (BMI).

## 4. Discussion

The study population comprised 70 patients diagnosed with lung carcinoma, predominantly males (75.7%), with a median age of 67.3 years. A significant majority of them (85.7%) were smokers, supporting previous data found in the literature, according to which smoking is a considerable risk factor for lung cancer. It induces inflammation by stimulating the production of cytokines, growth factors, and cell-derived reactive oxygen and nitrogen species, leading to DNA alterations [39].

Regarding the histopathological type of lung cancer, 64 patients from our study group were diagnosed with non-small cell lung carcinoma (NSCLC) and 6 with small cell lung carcinoma (SCLC). Within the NSCLC group, adenocarcinoma was the most prevalent subtype (51.6%), followed by squamous cell carcinoma (39.1%) and other types (9.3%). These findings are supported by previous studies, which stated that NSCLC is more frequently diagnosed among lung cancer patients, with adenocarcinoma being the most commonly diagnosed type of NSCLC, comprising approximately 40% of the NSCLC cases [40,41].

Most of our patients were diagnosed as having advanced stages of the disease: 55.7% at stage IV and 41.4% at stage III. This late-stage diagnosis is a persistent challenge in lung cancer management, often due to the asymptomatic nature of early disease and a lack of effective screening programs.

Sarcopenia, assessed by determining the skeletal muscle area (SMA) at the L3 vertebral level, was present in 48.6% of patients at diagnosis. Our study showed that sarcopenic patients were significantly older than non-sarcopenic patients and had lower levels of hemoglobin and hematocrit, suggesting a possible correlation between muscle wasting, anemia, and aging. These findings are supported by previous studies in which pre-treatment sarcopenia was significantly associated with low hemoglobin levels and an overall decreased survival and prognosis [42]. In addition, sarcopenic patients had a more advanced tumor stage (median: 4.0 vs. 3.0; *p* = 0.0104), older age (median: 70.0 vs. 65.0; *p* = 0.0177), and a higher severity score (median: 11.0 vs. 9.0; *p* = 0.0038) compared to those without sarcopenia. These findings reinforce that sarcopenic changes are associated with a more advanced disease stage at diagnosis, greater biological vulnerability, and a more advanced disease stage at diagnosis. When we evaluated the exact relationship between the SMA of the included patients and the most important clinical and biological parameters, we observed that SMA was negatively correlated with tumor stage, further suggesting that lower skeletal muscle mass can lead to a more aggressive carcinogenesis process. This emphasizes the importance of early nutritional and physical assessments in lung cancer management, as patients with sarcopenia can exhibit a decreased response to chemotherapeutic regimens and an increased drug toxicity response [43,44]. In addition, our study showed a positive correlation between SMA and Hb and Ht levels, showing that a higher skeletal muscle mass is associated with better hematological outcomes. These findings are in line with previous studies, which also stated that Hb levels might act as a negative prognostic factor in cancer patients, with anemia being associated with poor prognosis [45].

As inflammation has been cited in recent years as an essential part of carcinogenesis, we studied easily achievable CBC-derived biomarkers, similar to the variations in different cellular lines in lung cancer patients at the initial diagnosis. Our results showed that leukocyte count, monocyte count, and the neutrophil, eosinophil, and platelet counts were elevated at the time of initial diagnosis in our study population. Previous studies supported these findings, as neutrophilia and leukocytosis were linked to malignancy-associated chronic inflammation during tumoral growth and lysis [46,47,48]. In addition, high levels of neutrophils were linked with disease progression and an increased risk of distant metastasis. Regarding platelet count, high platelet levels were associated with poor overall survival in lung and colorectal cancer patients [49].

Furthermore, as seen in Table 3, all nine of the CBC-derived inflammatory markers included in our study presented elevated values at different stages of the disease, with an observed tendency for the number of patients with higher levels of inflammatory markers to increase as the disease severity progressed. In our study population, the SII was negatively correlated with age (ρ = −0.26, *p* = 0.0268), while both SII (ρ = −0.30, *p* = 0.0277) and SIRI (ρ = −0.28, *p* = 0.0445) were negatively correlated with BMI, suggesting that younger patients and those with a lower BMI tended to have higher inflammatory profiles. Therefore, our results align with previous studies that identified systemic inflammation as an essential contributor to carcinogenesis, as cancer is thought to develop more easily in tissues with chronic inflammatory processes [45,49,50,51].

In addition, the TNM stage at diagnosis, the histologic type of the lung carcinoma, and the degree of associated systemic inflammation and sarcopenia presence were all cited as potential predictors of severity in lung cancer patients; therefore, the development of a severity score that integrates all these essential characteristics represents an important step toward the early identification of high-risk lung cancer patients and a personalized treatment approach. The developed HISLIS severity score can effectively stratify patients by disease severity in three primary severity grades (low/medium/high), with higher severity grades being correlated with advanced tumor stages at diagnosis and elevated inflammatory markers. Our results showed that PLR (ρ = 0.74, *p* < 0.001), NLR (ρ = 0.70, *p* < 0.001), EMR (ρ = 0.30, *p* = 0.013), SIRI (ρ = 0.62, *p* < 0.001), MLR (ρ = 0.61, *p* < 0.001), AISI (ρ = 0.55, *p* < 0.001), d-NLR (ρ = 0.58, *p* < 0.001), SII (ρ = 0.46, *p* = 0.0001), tumor stage (ρ = 0.41, *p* = 0.0004), and the presence of sarcopenia (ρ = 0.36, *p* = 0.0024) were correlated with the severity grade. These findings are supported by previously published papers, which suggest that increased systemic inflammation and sarcopenic changes are strongly associated with greater severity [52,53,54,55,56]. The same findings were confirmed in our study population after applying the Kruskal–Wallis test to assess the distribution of selected clinical and inflammatory variables across the three predefined severity grades. The observed tendency was that multiple systemic inflammatory markers like NLR, d-NLR, MLR, PLR, SII, AISI, and SIRI were significantly higher in patients classified with more severe disease. These findings support the discriminative capacity of the proposed severity score and its association with clinical progression and systemic inflammation.

To our knowledge, this is the first study published in the literature that integrates these four main characteristics of lung tumors into a single severity score: HISLIS. Our study idea relied on the fact that integrating sarcopenia and inflammatory markers into a single severity score, one that accounts for associated tumor characteristics like histology and TNM grading, has important clinical implications. The HISLIS may allow clinicians to identify high-risk patients earlier and tailor interventions such as nutritional support or anti-inflammatory strategies. Furthermore, since CT scans are routinely available in cancer staging, including SMA at the L3 level, as part of the routine imaging assessment process, their use is practical and evidence-based [57].

Although we believe that the present study presents valuable information, it has its limitations. The primary limitation is the absence of follow-up data regarding the included patients, in addition to the retrospective nature of the study and the limited number of patients included. Therefore, we intend to strengthen the scientific value of the current research by conducting future prospective studies that contain additional information regarding patients’ survival, treatment response, and associated complications. The focus should be on validating the severity score in larger, prospective cohorts and correlating it with long-term outcomes, with the final goal of using the score as a screening tool for the early identification of high-risk lung cancer patients.

## 5. Conclusions

Lung cancer remains the malignancy that is associated with the highest rate of death worldwide. Identifying and integrating a complex score into the routine assessment of lung cancer patients that focuses on the main characteristics of the patients and the tumor itself represents a key step toward improving the early identification, risk classification, and personalized therapeutic approach for this highly deadly condition. The proposed HISLIS severity score integrates four main characteristics (systemic inflammation, CT scan-determined sarcopenic changes, TNM stage, and tumor histological type) as a reference point for the improved clinical and paraclinical assessment of lung carcinogenesis processes.

## Figures and Tables

**Table 1:** Formulas of the CBC-derived inflammatory biomarkers.

Parameter	Formula
Neutrophil-to-lymphocyte ratio (NLR)	Neutrophil count/lymphocyte count [×10^3^/μL] [34]
Derived-neutrophil-to-lymphocyte ratio (d-NLR)	Neutrophil count/(WBC − neutrophil count) [×10^3^/μL] [35]
Monocyte-to-lymphocyte ratio (MLR)	Monocyte count/lymphocyte count [×10^3^/μL] [35]
Platelet-to-lymphocyte ratio (PLR)	Platelet count/lymphocyte count [×10^3^/μL] [34]
Eosinophil-to-neutrophil ratio (ENR)	Eosinophil count/neutrophil count [×10^3^/μL] [36]
Eosinophil-to-monocyte ratio (EMR)	Eosinophil count/monocyte count [×10^3^/μL] [37]
Systemic inflammatory index (SII)	(Neutrophil count × platelet count)/lymphocyte count [×10^3^/μL] [38]
Systemic inflammatory response index (SIRI)	(Neutrophil count × monocyte count)/lymphocyte count [×10^3^/μL] [35]

**Table 2:** The components of the HISLIS score and the assigned score for each variable.

**Component**	**Variable Type**	**Assigned Score**
Sarcopenia	Binary categorical	1 point if present
TNM Stage	Ordinal (1–4)	Stage 1 = 0 points Stage 2 = 1 point Stage 3 = 2 points Stage 4 = 3 points
Histological Subtype	Encoded categorical (0–3)	SCLC = 2 points Squamous/other NSCLC = 1 point Adenocarcinoma = 0 points
CBC-derived inflammatory indexes	Binary categorical	1 point for each elevated marker

**Table 3:** Numerical distribution of patients with elevated levels of inflammatory markers and cellular line count values, based on the disease stage at diagnosis.

Parameter	Stage II		Stage III		Stage IV	
	Elevated Values	Normal Values	Elevated Values	Normal Values	Elevated Values	Normal Values
AISI	2	0	23	6	36	3
EMR	0	2	11	18	18	21
ENR	0	2	5	24	5	34
Eosinophils	0	2	2	27	5	34
Leukocytes	0	2	9	20	14	25
MLR	1	1	13	16	22	17
Monocytes	0	2	0	29	3	36
NLR	1	1	19	10	32	7
Neutrophils	0	2	10	19	20	19
PLR	1	1	17	12	28	11
SII	1	1	22	7	33	6
SIRI	1	1	19	10	33	6
Platelets	0	2	7	22	14	25
d-NLR	1	1	18	11	31	8

**Table 4:** Severity grade, based on tumor stage and the histological type of lung carcinoma.

Severity Grade	Tumor Stage	SCLC	Adenocarcinoma	Squamous Cell Carcinoma	Other NSCLC
Low	II	0	1	0	0
Low	III	1	5	4	2
Low	IV	1	5	1	0
Medium	III	0	3	5	0
Medium	IV	0	3	1	1
High	II	0	0	0	1
High	III	1	0	7	1
High	IV	3	16	7	1

**Table 5:** Main differences between sarcopenic and non-sarcopenic patients.

Variable	Median (Without Sarcopenia)	Median(With Sarcopenia)	*p*-Value	Difference Direction
BMI	24.3	23.84	0.1693	Higher without sarcopenia
SMA	138.5	99.0	0.0000	Higher without sarcopenia
Tumor Stage	3.0	4.0	0.0104	Higher with sarcopenia
Age	65.0	70.0	0.0177	Higher with sarcopenia
Severity Score	9.0	11.0	0.0038	Higher with sarcopenia
NLR	4.19	6.62	0.1169	Higher with sarcopenia
d-NLR	2.89	3.29	0.2122	Higher with sarcopenia
PLR	237.98	276.19	0.6249	Higher with sarcopenia
SII	1670.5	2308.06	0.2959	Higher with sarcopenia
SIRI	2.39	3.6	0.1056	Higher with sarcopenia

## Data Availability

No new data were created or analyzed in this study. Data sharing is not applicable to this article.

## Abbreviations

The following abbreviations are used in this manuscript:

COPD	chronic obstructive pulmonary disorder
SCLC	small cell lung carcinoma
NSCLC	non-small cell lung carcinoma
EGFR	epidermal growth factor
BIA	bioelectrical impedance analysis
okDXA	dual-energy X-ray absorptiometry
CT scans	computed tomography scans
SMA	skeletal muscle area
L3SMA	skeletal muscle area determined at the third lumbar vertebra level
CBC	complete blood count
NLR	neutrophil-to-lymphocyte ratio
d-NLR	derived neutrophil-to-lymphocyte ratio
MLR	monocyte-to-lymphocyte ratio
PLR	platelet-to-lymphocyte ratio
ENR	eosinophil-to-neutrophil ratio
EMR	eosinophil-to-monocyte ratio
SII	systemic inflammatory index
SIRI	systemic inflammatory response index
AISI	aggregate index of systemic inflammation
BMI	body mass index
TNM	tumor, node, metastasis
HISLIS	histology, sarcopenia, and lung inflammation score
NOS	not otherwise specified
P 33	33rd percentile
P 66	66th percentile
Hb	Hemoglobin
Ht	Hematocrit

## References

[1] Kratzer T.B., Bandi P., Freedman N.D., Smith R.A., Travis W.D., Jemal A., Siegel R.L. (2024). Lung cancer statistics, 2023. Cancer.

[2] Midha A., Dearden S., McCormack R. (2015). EGFR mutation incidence in non-small-cell lung cancer of adenocarcinoma histology: A systematic review and global map by ethnicity (mutMapII). Am. J. Cancer Res..

[3] WHO (2018). WHO Report on the Global Tobacco Epidemic, 2017.

[4] Benusiglio P.R., Fallet V., Sanchis-Borja M., Coulet F., Cadranel J. (2021). Lung cancer is also a hereditary disease. Eur. Respir. Rev..

[5] Chao L., Shaoyuan L., Li D., Yan X., Xiaonan W., Hui W., Zijin Z., Ting G., Yongqiang Z., Lin L. (2023). Global burden and trends of lung cancer incidence and mortality. Chin. Med. J..

[6] Brenner D.R., Boffetta P., Duell E.J., Bickeboller H., Rosenberger A., McCormack V., Muscat J.E., Yang P., Wichmann H.-E., Brueske-Hohlfeld I. (2012). Previous Lung Diseases and Lung Cancer Risk: A Pooled Analysis from the International Lung Cancer Consortium. Am. J. Epidemiol..

[7] Brenner D.R., McLaughlin J.R., Hung R.J. (2011). Previous Lung Diseases and Lung Cancer Risk: A Systematic Review and Meta-Analysis. PLoS ONE.

[8] Ang L., Ghosh P., Seow W.J. (2021). Association between previous lung diseases and lung cancer risk: A systematic review and meta-analysis. Carcinogenesis.

[9] Khusnurrokhman G., Wati F.F. (2022). Tumor-promoting inflammation in lung cancer: A literature review. Ann. Med. Surg..

[10] Han J.Y., Park K., Kim S.W., Lee D.H., Kim H.Y., Kim H.T., Ahn M.J., Yun T., Ahn J.S., Suh C. (2012). First-SIGNAL: First-Line Single-Agent Iressa Versus Gemcitabine and Cisplatin Trial in Never-Smokers with Adenocarcinoma of the Lung. J. Clin. Oncol..

[11] Maemondo M., Inoue A., Kobayashi K., Sugawara S., Oizumi S., Isobe H., Gemma A., Harada M., Yoshizawa H., Kinoshita I. (2010). Gefitinib or Chemotherapy for Non–Small-Cell Lung Cancer with Mutated EGFR. N. Engl. J. Med..

[12] Ramalingam S.S., Vansteenkiste J., Planchard D., Cho B.C., Gray J.E., Ohe Y., Zhou C., Reungwetwattana T., Cheng Y., Chewaskulyong B. (2020). Overall Survival with Osimertinib in Untreated, EGFR-Mutated Advanced NSCLC. N. Engl. J. Med..

[13] Kara M., Kaymak B., Frontera W., Ata A.M., Ricci V., Ekiz T., Chang K., Han D.-S., Michail H., Quittan M. (2021). Diagnosing sarcopenia: Functional perspectives and a new algorithm from the ISarcoPRM. J. Rehabil. Med..

[14] Santilli V., Bernetti A., Mangone M., Paoloni M. (2014). Clinical definition of sarcopenia. Clin. Cases Min. Bone Metab..

[15] Cruz-Jentoft A.J., Bahat G., Bauer J., Boirie Y., Bruyère O., Cederholm T., Cooper C., Landi F., Rolland Y., Sayer A.A. (2019). Sarcopenia: Revised European consensus on definition and diagnosis. Age Ageing.

[16] Han D.S., Wu W.T., Hsu P.C., Chang H.C., Huang K.C., Chang K.V. (2021). Sarcopenia is associated with increased risks of rotator cuff tendon diseases among community-dwelling elders: A cross-sectional quantitative ultrasound study. Front. Med..

[17] Collins J., Noble S., Chester J., Coles B., Byrne A. (2014). The assessment and impact of sarcopenia in lung cancer: A systematic literature review. BMJ Open.

[18] Nishimura J.M., Ansari A.Z., D’Souza D.M., Moffatt-Bruce S.D., Merritt R.E., Kneuertz P.J. (2019). Computed tomography-assessed skeletal muscle mass as a predictor of outcomes in lung cancer surgery. Ann. Thorac. Surg..

[19] Kawaguchi Y. (2023). The process to overcome lung cancer sarcopenia. J. Thorac. Dis..

[20] Kawaguchi Y., Watanabe A., Shiratori T., Kaku R., Ueda K., Okamoto K., Kataoka Y., Ohshio Y., Hanaoka J. (2024). Myostatin expression in lung cancer induces sarcopenia and promotes cancer progression. Gen. Thorac. Cardiovasc. Surg..

[21] Nam K., Lee J.Y., Ko Y., Kim K.W., Lee H.-S., Hong S.W., Park J.H., Hwang S.W., Yang D.-H., Ye B.D. (2023). Impact of sarcopenia on clinical course of inflammatory bowel disease in Korea. Dig. Dis. Sci..

[22] Nie X., Zou M., Song C., Zhang P., Ma D., Cui D., Cheng G., Li L. (2025). Survival impact and risk factors of skeletal muscle loss during first-line EGFR-TKIs therapy in advanced lung adenocarcinoma patients. BMC Cancer.

[23] Mountzios G., Samantas E., Senghas K., Zervas E., Krisam J., Samitas K., Bozorgmehr F., Kuon J., Agelaki S., Baka S. (2021). Association of the advanced lung cancer inflammation index (ALI) with immune checkpoint inhibitor efficacy in patients with advanced non-small-cell lung cancer. ESMO Open.

[24] Lenci E., Cantini L., Pecci F., Cognigni V., Agostinelli V., Mentrasti G., Lupi A., Ranallo N., Paoloni F., Rinaldi S. (2021). The gustave roussy immune (GRIm)-score variation is an early-on-treatment biomarker of outcome in advanced non-small cell lung cancer (NSCLC) patients treated with first-line pembrolizumab. J. Clin. Med..

[25] Ali W.A.S., Hui P., Ma Y., Wu Y., Zhang Y., Chen Y., Hong S., Yang Y., Huang Y., Zhao Y. (2021). Determinants of survival in advanced non-small cell lung cancer patients treated with anti-PD-1/PD-L1 therapy. Ann. Transl. Med..

[26] Peng L., Wang Y., Liu F., Qiu X., Zhang X., Fang C., Quian X., Li Y. (2020). Peripheral blood markers predictive of outcome and immune-related adverse events in advanced non-small cell lung cancer treated with PD-1 inhibitors. Cancer Immunol. Immunother..

[27] Cederholm T., Compher C., Correia M.I.T.D., Gonzalez M.C., Fukushima R., Higashiguchi T., Van Gossum A., Jensen G.L. (2019). Response to the Letter: Comment on “GLIM Criteria for the Diagnosis of Malnutrition—A Consensus Report from the Global Clinical Nutrition Community”. Some Considerations about the GLIM Criteria—A Consensus Report for the Diagnosis of Malnutrition by Drs. LB Da Silva Passos and DA De-Souza. Clin. Nutr..

[28] Mariean C.R., Tiucă O.M., Mariean A., Cotoi O.S. (2023). Cancer Cachexia: New Insights and Future Directions. Cancers.

[29] Charrière K., Ragusa A., Genoux B., Vilotitch A., Artemova S., Dumont C., Beaudoin P.A., Madiot P.E., Ferretti R., Bricault I. (2024). ODIASP: An Open User-Friendly Software for Automated SMI Determination—Application to an Inpatient Population. medRxiv.

[30] Magudia K., Bridge C.P., Bay C.P., Babic A., Fintelmann F.J., Troschel F.M., Miskin N., Wrobel W.C., Brais L.K., Andriole K.P. (2021). Population-Scale CT-based Body Composition Analysis of a Large Outpatient Population Using Deep Learning to Derive Age-, Sex-, and Race-specific Reference Curves. Radiology.

[31] Bridge C.P., Rosenthal M., Wright B., Kotecha G., Fintelmann F., Troschel F., Miskin N., Desai K., Wrobel W., Babic A., Stoyanov D., Taylor Z., Sarikaya D., McLeod J., González Ballester M.A., Codella N.C.F., Martell A., Maier-Hein L.,  Malpani A., Zenati M.A. (2018). Fully-Automated Analysis of Body Composition from CT in Cancer Patients Using Convolutional Neural Networks. OR 2.0 Context-Aware Operating Theaters, Computer Assisted Robotic Endoscopy, Clinical Image-Based Procedures, and Skin Image Analysis (CARE 2018, CLIP 2018, OR 2.0 2018, ISIC 2018): Proceedings.

[32] Paris M.T., Tandon P., Heyland D.K., Furberg H., Premji T., Low G., Mourtzakis M. (2020). Automated body composition analysis of clinically acquired computed tomography scans using neural networks. Clin. Nutr..

[33] Derstine B.A., Holcombe S.A., Ross B.E., Wang N.C., Su G.L., Wang S.C. (2018). Skeletal muscle cutoff values for sarcopenia diagnosis using T10 to L5 measurements in a healthy US population. Sci. Rep..

[34] Mandaliya H., Jones M., Oldmeadow C., Nordman I.I.C. (2019). Prognostic biomarkers in stage IV non-small cell lung cancer (NSCLC): Neutrophil to lymphocyte ratio (NLR), lymphocyte to monocyte ratio (LMR), platelet to lymphocyte ratio (PLR) and advanced lung cancer inflammation index (ALI). Transl. Lung Cancer Res..

[35] Detterbeck F.C. (2018). The eighth edition TNM stage classification for lung cancer: What does it mean on main street?. J. Thorac. Cardiovasc. Surg..

[36] Guo B., Liu X., Si Q., Zhang D., Li M., Li X., Zhao Y., Hu F., Zhang M., Liu Y. (2024). Associations of CBC-Derived inflammatory indicators with sarcopenia and mortality in adults: Evidence from Nhanes 1999–2006. BMC Geriatr..

[37] Wei J., Brown L., Gao B., Nagrial A., da Silva I.P. (2023). P1.21-08 Eosinophil to Neutrophil Ratio Predicts Efficacy in Patients Receiving Immunotherapy in Metastatic Non-small Cell Lung Cancer (mNSCLC). J. Thorac. Oncol..

[38] Dubinett S.M. (2015). Inflammation and Lung Cancer.

[39] Engels E.A. (2008). Inflammation in the development of lung cancer: Epidemiological evidence. Expert. Rev. Anticancer Ther..

[40] Zappa C., Mousa S.A. (2016). Non-small cell lung cancer: Current treatment and future advances. Transl. Lung Cancer Res..

[41] ALQudah M.A., ALFaqih M.A., Hamouri S., Al-Shaikh A.F., Haddad H.K., Al-Quran W.Y., Alebbini M.M., Amer N.B., AL-Smadi H.I., Alzoubi K.H. (2021). Epidemiology and histopathological classification of lung cancer: A study from Jordan, retrospective observational study. Ann. Med. Surg. [Internet].

[42] Resende L.S.D.A., Amorim F.V.D., Conceição M.S., Jales R.M., Pereira P.N., Sarian L.O., Baiocchi G., Derchain S., da Silva Filho A.L. (2024). Sarcopenia and Anemia Are Predictors of Poor Prognostic in Cervical Cancer Patients. Open J. Obstet. Gynecol..

[43] Bozzetti F. (2017). Forcing the vicious circle: Sarcopenia increases toxicity, decreases response to chemotherapy and worsens with chemotherapy. Ann. Oncol..

[44] Chindapasirt J. (2016). Sarcopenia in Cancer Patients. Asian Pac. J. Cancer Prev..

[45] Caro J.J., Salas M., Ward A., Goss G. (2001). Anemia as an independent prognostic factor for survival in patients with cancer: A systemic, quantitative review. Cancer.

[46] Mazzella A., Maiolino E., Maisonneuve P., Loi M., Alifano M. (2023). Systemic Inflammation and Lung Cancer: Is It a Real Paradigm? Prognostic Value of Inflammatory Indexes in Patients with Resected Non-Small-Cell Lung Cancer. Cancers.

[47] Łochowski M., Chałubińska-Fendler J., Zawadzka I., Łochowska B., Rębowski M., Brzeziński D., Kozak J. (2021). The Prognostic Significance of Preoperative Platelet-to-Lymphocyte and Neutrophil-to-Lymphocyte Ratios in Patients Operated for Non-Small Cell Lung Cancer. Cancer Manag. Res..

[48] Şahin F., Aslan A.F. (2018). Relationship between Inflammatory and Biological Markers and Lung Cancer. J. Clin. Med..

[49] Jurasz P., Alonso-Escolano D., Radomski M.W. (2004). Platelet–cancer interactions: Mechanisms and pharmacology of tumour cell-induced platelet aggregation. Br. J. Pharmacol..

[50] Cho W.C., Kwan C.K., Yau S., So P.P., Poon P.C., Au J.S. (2011). The role of inflammation in the pathogenesis of lung cancer. Expert. Opin. Ther. Targets.

[51] Hu B., Yang X.R., Xu Y., Sun Y.F., Sun C., Guo W., Zhang X., Wang W.-M., Qiu S.-J., Zhou J. (2014). Systemic Immune-Inflammation Index Predicts Prognosis of Patients after Curative Resection for Hepatocellular Carcinoma. Clin. Cancer Res..

[52] Choi J.E., Villarreal J., Lasala J., Gottumukkala V., Mehran R.J., Rice D., Yu J., Feng L., Cata J.P. (2015). Perioperative neutrophil:lymphocyte ratio and postoperative NSAID use as predictors of survival after lung cancer surgery: A retrospective study. Cancer Med..

[53] Glasner A., Avraham R., Rosenne E., Benish M., Zmora O., Shemer S., Meiboom H., Ben-Eliyahu S. (2010). Improving survival rates in two models of spontaneous postoperative metastasis in mice by combined administration of a beta-adrenergic antagonist and a cyclooxygenase-2 inhibitor. J. Immunol..

[54] Sarraf K.M., Belcher E., Raevsky E., Nicholson A.G., Goldstraw P., Lim E. (2009). Neutrophil/lymphocyte ratio and its association with survival after complete resection in non-small cell lung cancer. J. Thorac. Cardiovasc. Surg..

[55] Zhai B., Chen J., Wu J., Yang L., Guo X., Shao J., Xu H., Shen A. (2021). Predictive value of the hemoglobin, albumin, lymphocyte, and platelet (HALP) score and lymphocyte-to-monocyte ratio (LMR) in patients with non-small cell lung cancer after radical lung cancer surgery. Ann. Transl. Med..

[56] Yang N., Han X., Yu J., Shu W., Qiu F., Han J. (2020). Hemoglobin, albumin, lymphocyte, and platelet score and neutrophil-to-lymphocyte ratio are novel significant prognostic factors for patients with small-cell lung cancer undergoing chemotherapy. J. Cancer Res. Ther..

[57] Martin L., Birdsell L., MacDonald N., Reiman T., Clandinin M.T., McCargar L.J., Murphy R., Ghosh S., Sawyer M.B., Baracos V.E. (2013). Cancer Cachexia in the Age of Obesity: Skeletal Muscle Depletion Is a Powerful Prognostic Factor, Independent of Body Mass Index. J. Clin. Oncol..

